# Floral intra‐individual variation differs between morphs and is linked to pollen deposition in self‐incompatible distylous plants

**DOI:** 10.1111/plb.70299

**Published:** 2026-09-25

**Authors:** R. Trevizan, V. Ferrero, F. Telles, L. Oliveira, L. Melo, F. W. Amorim, P. E. Oliveira

**Affiliations:** ^1^ Programa de Pós‐Graduação em Biologia Vegetal Universidade Estadual de Campinas Campinas Brazil; ^2^ Programa de Pós‐Graduação em Ecologia, Conservação e Manejo da Fauna Silvestre Universidade Federal de Minas Gerais Belo Horizonte Brazil; ^3^ Department of Biodiversity and Environmental Management, Campus de Vegazana University of León León Spain; ^4^ Instituto de Biologia Universidade Federal de Uberlândia, Campus Umuarama Uberlândia Minas Gerais Brazil; ^5^ São Paulo State University (UNESP), Institute of Biosciences Rio Claro Brazil; ^6^ Department of Ecology and Genetics, Evolutionary Biology Centre Uppsala University Uppsala Sweden; ^7^ Laboratório de Ecologia da Polinização e Interações ‐ LEPI, Departamento de Biodiversidade e Bioestatística, Instituto de Biociências Universidade Estadual Paulista “Júlio de Mesquita Filho” (UNESP) Botucatu Brazil

**Keywords:** Bees, floral traits, hummingbirds, local adaptation, *Palicourea rigida*, reproductive success, within‐individual variation

## Abstract

- Flowers are expected to exhibit lower trait variability than vegetative traits, thereby enhancing precise pollination and reproductive success, especially in self‐incompatible species that rely on cross‐pollination. This is particularly important in distylous plants, which have two co‐dependent flower morphs with reciprocal positioning of sexual structures (L‐ and S‐morph). However, the influence of intra‐individual floral variation on the reproductive process has often been neglected, even though this variation underlies selection for precise floral adjustment.
- This study investigated the intra‐individual variation in floral traits across different morphs and populations of distylous plants with contrasting pollinator guilds (bees and hummingbirds), focusing on the implications of this variation for reproductive success and floral resource features.
- We showed intra‐individual variation differences between morphs and populations in stigma length and stigma height, which may reflect distinct pollination pressure across populations. Contrastingly, the similar levels of variation are maintained across morphs, potentially facilitating effective pollen transfer within these self‐incompatible distylous populations. Intra‐individual variation in corolla length was positively associated with total pollen deposition, which was also morph‐dependent (greater in L‐morph flowers in the bee‐pollinated population). However, intra‐individual variation did not affect fruit set.
- Our results provide a novel perspective on the effect of intra‐individual variation in the reproductive process of distylous plants, indicating that such variation might serve as a strategy to occupy a greater positional range accessed by distinct pollinators, which would increase the chances of cross‐pollen deposition among flowers within individuals.

## Graphical abstract

For most traits, floral intra‐individual variation was similar between morphs and populations with different pollinator guilds (bees and hummingbirds), potentially facilitating effective pollen transfer in distylous systems. Still, greater variation in corolla length increased pollen deposition in L‐morph flowers in the bee‐pollinated population.

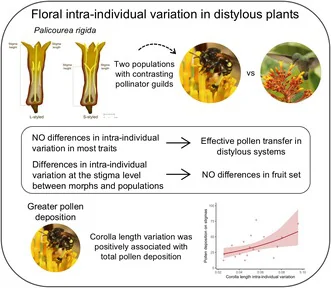

## INTRODUCTION

Floral trait variation, at both the individual and population levels, influences pollinator visitation to animal‐pollinated plants, serving as a crucial factor in determining plant reproductive fitness (Fenster *et al*. 2004; Harder & Johnson 2009). Due to their role in reproduction, flowers are expected to exhibit lower variability than vegetative traits (Armbruster *et al*. 2009, 2014; Herrera 2009). This expectation is closely associated with the concept of pollination accuracy, which refers to the consistency and spatial alignment with which pollen is removed from and deposited onto functionally optimal positions on pollinators and stigmas, a condition expected to improve the fit between pollinators and floral traits (Berg 1960; Armbruster *et al*. 2009, 2014). Accordingly, even lower variability in floral traits may be expected in species with a self‐incompatibility system, which rely exclusively on precise cross‐pollination and cross‐fertilization to produce fruits and seeds (Ganders 1979; Ordano *et al*. 2008; Delgado‐Dávila *et al*. 2016).

Environmental factors can independently impact different plant individuals, leading to variation in the development, structure and physiology of repeated plant units, such as leaves, flowers, fruits and seeds at the plant level (Herrera 2009, 2017; Diggle 2014; Møller *et al*. 2023). Such environmentally driven variation may occur not only among individuals, but also within individuals, generating phenotypic differences among repeated floral units produced by the same plant (Herrera 2009, 2017; Diggle 2014). When variations occur in flower reproductive traits, such as stamens and/or stigmas or floral structures guiding or restricting pollinator entry, such as the corolla tube, it can influence the pollination process in different ways (Dai *et al*. 2016; Liu *et al*. 2016; Yuan *et al*. 2017; Romero‐Bravo & Castellanos 2024). For example, the reduction of herkogamy (*i.e*. spatial separation between anthers and stigma within a flower) can lead to increased sexual interference and potential disruptions in the pollination process, affecting the transfer of conspecific pollen and/or the production of viable seeds, as well as clogging the stigma with self‐pollen (Webb & Lloyd 1986; Keller *et al*. 2014; Barranco *et al*. 2019; Navarro *et al*. 2021). More broadly, variation in other floral traits that influence pollinator attraction, handling or contact with sexual organs has been found to affect plant fitness at the population level, such as floral display (Mitchell *et al*. 2004; Cunha & Aizen 2023), flower colour (Brito *et al*. 2015) and flower morphology (Eisen *et al*. 2023).

Despite its potential significance for plant fitness, intra‐individual variation, defined as variation in a trait within an individual plant, remains poorly studied in floral traits of pollination‐dependent systems (Herrera 2009; Pinto *et al*. 2022). Nevertheless, existing evidence indicates that intra‐individual variation affects plant fitness, with variation in floral traits enhancing female reproductive success (Arceo‐Gómez *et al*. 2017) and male reproductive success (Paglia *et al*. 2023). In some cases, intra‐individual floral variation may produce contrasting or context‐dependent effects on plant reproduction (Dai *et al*. 2016). In *Passiflora incarnata* (Passifloraceae), style deflexion varies both within flowers through time and within individuals (*i.e*. among flowers of the same plant). Lower deflexion and reduced variation in that deflexion increased pollen deposition, which in turn increased seed number. In contrast, higher deflexion and greater variability of that deflexion increased variation in pollen deposition, which resulted in heavier seeds, possibly because variation in style position increases the diversity of pollen donors and potentially improves pollen quality (Dai *et al*. 2016). These contrasting outcomes highlight that intra‐individual variation in floral traits may involve trade‐offs between pollination precision and reproductive assurance. This underscores the need to understand the ecological implications of floral trait variation at the individual level, especially in self‐incompatibility systems, such as those associated with floral polymorphisms.

Distyly polymorphism is considered a reproductive strategy promoting cross‐pollination. Distylous populations present individuals with two floral morphs with reciprocal spatial separation of anthers and styles: short‐styled flowers (hereafter S‐styled), with stigma below the anthers' height and long‐styled flowers (hereafter L‐styled), with stigma above the height of the anthers (Darwin 1877). The correspondence between the reproductive parts of different morphs (*i.e*. reciprocal herkogamy) facilitates accurate pollen transfer between floral morphs, thereby bolstering cross‐pollination and achieving an optimal seed set in populations (Darwin 1877; Keller *et al*. 2014; Trevizan *et al*. 2021b). Furthermore, given the connection of this morphological polymorphism to a self‐ and intramorph‐incompatibility system, the high accuracy of sexual floral structures is essential to ensure the fit between pollinators and flowers (Barrett & Shore 2008; Delgado‐Dávila *et al*. 2016). This precision minimizes interference between male and female functions, increasing the likelihood of compatible pollen deposition (Barrett & Shore 2008; Trevizan *et al*. 2021b). Even slight differences in floral morphology can significantly affect the pollination efficiency of distylous flowers (Ganders 1979; Faivre & McDade 2001). Thus, selection may result in a more precise correspondence between morphs, leading to well‐defined reciprocal herkogamy and disassortative pollination (Keller *et al*. 2014).

Variations in floral structures have been documented within individual distylous populations (Pinto *et al*. 2022; Trevizan *et al*. 2024a). However, the consequences of intra‐individual variation for reproductive success remain largely underexplored in pollinator‐dependent systems, such as distyly. Since distylous plants rely on precise pollen transfer between morphs (L‐styled and S‐styled), they are valuable models for investigating how floral morphological variations might impact reproductive success. Moreover, because distylous species can be pollinated by different animal groups, selective pressures and patterns of morphological variation may vary in response to contrasting pollination systems (Trevizan *et al*. 2021a). Distylous Rubiaceae species, such as *Palicourea rigida*, are pollinated by a diverse assemblage of vectors in Brazilian savanna ecosystems, ranging from bees to hummingbirds, and these distinct pollinators may lead to different levels of morphological variation among populations.

Based on this idea, our study aimed to investigate intra‐individual variation in floral traits between morphs of distylous populations characterized by distinct life histories and pollinator guilds, i.e., one dominated by bees and the other by hummingbirds. Additionally, we investigated the implications of intraspecific variation for different components of reproductive success and floral resource properties. We specifically focused on three questions: (1) Does intra‐individual variation differ between morphs and populations? (2) Does intra‐individual variation influence aspects of reproductive success (*i.e*. pollen deposition on stigmas, pollen release from anthers, and fruit set)? (3) Does intra‐individual variation influence aspects of resource properties (*i.e*. nectar calories)? Considering that pollinator‐mediated selection generally increases the fit between flowers and pollinators, we expected less intra‐individual variation between morphs within self‐incompatible populations, but differences between populations, given that different pollinator groups can exert distinct selective pressures. We also expected greater reproductive success to be associated with lower intra‐individual variation, reflecting a stronger fit between pollinators and flowers.

## MATERIALS AND METHODS

### Study sites

The study was conducted in two Cerrado areas: Caldas Novas State Park, in Caldas Novas, Goiás, Brazil (PESCAN) and Botucatu State Forest, in Botucatu, São Paulo, Brazil (BSF). PESCAN spans an area of 12,300 hectares (17°47′S; 48°40′W; altitude: 1000 m; Fig. S1) and is dominated by the Cerrado biome, the Brazilian Neotropical Savanna plant formations, ranging from open grasslands with scattered shrubs to typical *sensu stricto* Cerrado with small, contorted trees and also including gallery forests and palm swamp areas. On the other hand, BSF is a Cerrado remnant covering an area of 34 ha (22°55′S; 048°27′W; altitude: 850 m; Fig. S1). It predominantly features open grasslands with scattered shrubs and trees called locally Campo Sujo (Pilon *et al*. 2017, Ballarin *et al*. 2026). The climate of both regions is classified as Aw (Tropical Savanna climate with dry winters), with warmer and rainy summers (October–March) and cooler and dry winters (April–September) according to the Köppen–Geiger classification (Alvares *et al*. 2013; Franco *et al*. 2023).

### Study system

We studied two distylous populations of *Palicourea rigida* Kunth (Rubiaceae), representing contrasting floral morphological and pollination contexts. In the PESCAN area, the population is predominantly composed of shrubs or treelets (Fig. 1A), averaging 1.73 meters and is widely distributed in *sensu stricto* Cerrado on a plateau at approximately 1047 meters above sea level (Machado *et al*. 2010; Cardoso *et al*. 2022). The plant individuals display terminal inflorescences with tubular, orange flowers (Fig. 1B), with an average of 200 flowers produced per plant and lasting 1 day (Machado *et al*. 2010). The population is self‐incompatible and intramorph‐incompatible, and hummingbirds are the primary pollinators (Fig. 1C), with distinct species recorded visiting the flowers (Machado *et al*. 2010; Justino *et al*. 2012; Amorim 2020). In contrast, in the BSF, the population comprises plants growing as hemicryptophytes (*i.e*. small shrubs with the main trunk entirely underground). These plants are characterized by reduced stature, averaging 0.25 meters in height (Fig. 1D), and by individuals bearing inflorescences with conspicuous, markedly yellow flowers (Fig. 1E), with an average of 151 flowers per plant, lasting 1 day. The main pollinators are medium‐ to large‐bodied bees (Doria 2020; Fig. 1F). In both populations, the flowering period occurred during the rainy season, towards the end of the year (Machado *et al*. 2010; Cardoso *et al*. 2022). These marked differences in plant size, floral colour and pollinator guilds suggest distinct selective environments for floral traits between populations. Samples were collected for the PESCAN population from October to December 2020 and the BSF population in December 2020 and January 2021.

**Fig. 1:**
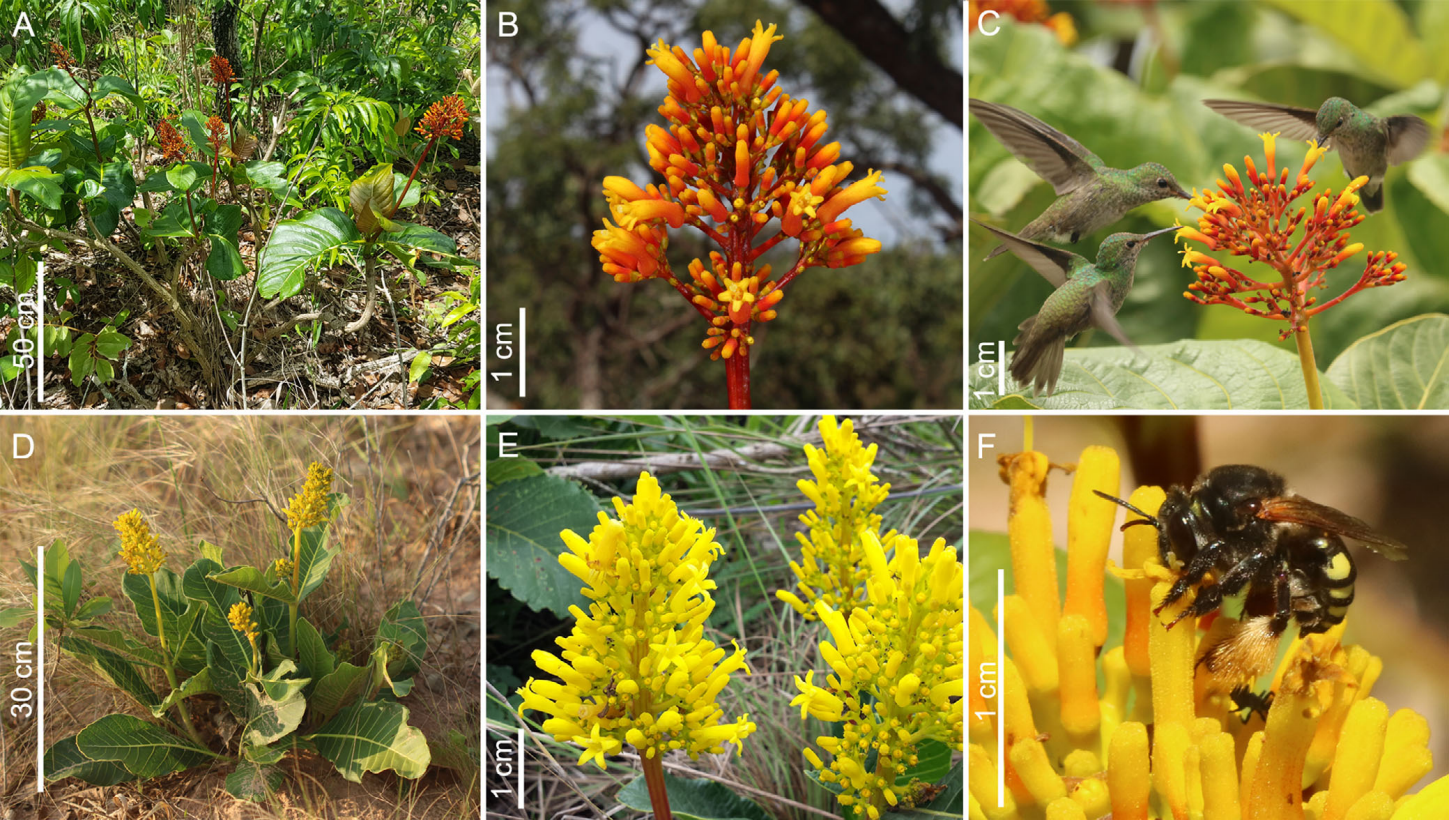
Morphological variation between populations of *Palicourea rigida* (Rubiaceae). Panels (A–C) correspond to the population from the Caldas Novas State Park (PESCAN), and (D–F) to the population from the Botucatu State Forest (BSF). In the PESCAN area, plants exhibit a typical shrub life form (A) bearing orange flowers (B) pollinated by hummingbirds (C). Panel (C) is a composite image of three photographs merged in Photoshop, showing the same individual of the hummingbird *Chionomesa fimbriata* visiting different flowers within the same inflorescence. In contrast, the BSF population comprises plants growing as hemicryptophytes (D) with conspicuous yellow flowers (E) pollinated by medium‐ to large‐bodied bees, such as *Epicharis iheringi* (F).

### Flower sampling procedures

To assess floral morphology and aspects of reproductive success, we used the same sampled flowers. Flowers were exposed to floral visitors to allow natural pollination. Because pollinator visits begin early in the morning (Machado *et al*. 2010), data collection started at 11:00 AM, after approximately 5 h of exposure to floral visitors. Following this exposure period, the flowers were collected, stored in a thermal box and transported to the laboratory for subsequent procedures (see below).

### Floral morphology measurements

We collected floral morphological traits from selected flowers across different individuals in each study area, PESCAN and BSF. In total, we collected 410 flowers, with five flowers per individual: 235 flowers from 47 individuals in PESCAN (23 S‐styled and 24 L‐styled) and 175 flowers from 35 individuals in BSF (17 S‐styled and 18 L‐styled). The five flowers were sampled from a single inflorescence per individual, ensuring standardized sampling effort across all individuals. The collected flowers were carefully dissected and positioned on a frame lined with millimetre‐scale graph paper and covered loosely with a glass plate. We took pictures with a camera (Canon Ti3 Eos; Lens Ef‐s 18‐55 mm), and measurements were subsequently taken using *ImageJ* software (Schneider *et al*. 2012). We measured the following morphological traits: (1) corolla length (from the base of the corolla to the opening of the petals); (2) stigma length; (3) stigma height (from the corolla base to the middle portion of the stigmatic lobes); (4) anther length; and (5) anther height (from the corolla base to the middle of the anthers; Fig. S2).

### Pollen deposition on stigmas

We quantified the total number of pollen grains deposited on the L‐styled and S‐styled stigmas as a measure of female fitness. Pistils were removed from the collected flowers and transferred to glass slides. Subsequently, stigmas were mounted in fuchsin jelly to preserve the pollen grains for subsequent counting. Pollen grains were counted using a microscope (Zeiss Axioplan).

### Pollen released from the anthers of flowers

We quantified the number of pollen grains remaining in the anthers as a measure of male pollination performance (Opedal *et al*. 2023) in L‐styled and S‐styled plants from both study populations. We collected three anthers from each flower previously used for morphology measurements and pollen deposition analyses to estimate the amount of pollen remaining after 5 h of exposure to natural pollinators. Anthers were stored in Eppendorf tubes containing 70% ethanol. In the laboratory, anthers were macerated and then vortexed for 60 s in 200 μl of a 70% ethanol–glycerol solution (3:1; adapted from Lloyd 1972). From this suspension, 10 μl were transferred to a Neubauer counting chamber for pollen grain counts under a light microscope to count pollen grains. The total number of pollen grains remaining per flower was estimated by scaling the counted pollen to the total solution volume and chamber volume (calculated as 200 μL × total pollen/chamber volume = 0.4) (Trevizan *et al*. 2023). We used these values to estimate the total pollen remaining in the sampled anthers and the total pollen per flower, assuming five anthers per flower.

To estimate pollen production per flower, pre‐anthesis floral buds from the same individuals were collected and processed using the same extraction protocol. Pollen removal during floral visitation was estimated by comparing the total number of pollen grains in floral buds with the number remaining in the anthers after pollinator exposure (see Statistical analyses for details).

### Fruit set

We counted the total number of flowers per individual plant, including both buds, open flowers and floral scars in the inflorescences (Machado *et al*. 2010), and monitored fruit production to assess fruit set. We excluded flowers collected for morphological and nectar analyses, as well as floral buds collected for pollen production estimation. A total of 81 individuals were included in the analysis: 47 from PESCAN (24 L and 23 B) and 34 from BSF (18 L and 16 B).

### Intra‐individual variation and floral resource features

Nectar production was measured in flowers previously bagged at the bud stage to prevent access by floral visitors (five flowers per plant, for a total of 305 flowers from 33 individuals in PESCAN and 27 individuals in BSF). We collected flowers between 10:00 AM and 12:00 PM. The nectar volume was extracted using capillary tubes (20 μL), and sugar concentration was measured with a hand refractometer (Galetto & Bernardello 2005). The amount of reward (calories) secreted by each flower was estimated using the following equation: y = 0.00226 + (0.00937x) + (0.0000585x^2^), where ‘*x’* is the refractometer reading and ‘*y’* is the mg of solute per μl (Galetto & Bernardello 2005). The total sugar (mg) secreted by each flower was obtained by multiplying ‘*y’* by the total volume (Galetto & Bernardello 2005). Morphological measurements were conducted on the same flowers used for nectar sampling to assess whether total sugar (mg) and energy content were associated with intra‐individual variation. Measurements were conducted using the methodology described in the ‘Floral morphology measurements *’* section.

### Data analyses

We initially explored intra‐individual variation and differences between morphs and populations. For each individual in each population, intra‐individual variation was quantified as the coefficient of variation (CV) for each trait, calculated as the standard deviation of measurements across the five flowers sampled from that individual, divided by the mean of that trait across those five flowers (March‐Salas *et al*. 2021). Specifically, we assessed intra‐individual variation in corolla length, stigma length, stigma height, anther length and anther height between L‐styled and S‐styled morphs and between populations. Subsequently, we fitted a generalized linear model (GLM) with a *Gamma* distribution and *log* link function, except for stigma height (as indicated in Table S1). In these models, each floral morphological trait CV was treated as a response variable, with morph, population and their interaction as predictor variables (Table S1).

After assessing differences in intra‐individual variation between morphs and populations, we evaluated the effects of this variation on aspects of reproductive success. Specifically, we focused on pollen deposition on stigmas, pollen release from anthers and the proportion of fruit set. We first examined variation in pollen deposition on stigmas between morphs and populations using a GLM with a *Gamma* distribution and *log* link function. The response variable was relative pollen deposition, calculated as the ratio of the total number of pollen grains deposited on a stigma to its length in millimetres (*i.e*. the number of pollen grains/stigma length; Trevizan *et al*. 2024b). Predictor variables included the morph, population and their interaction.

Following this analysis, we further tested whether pollen deposition was influenced by intra‐individual variation using additional models. We fitted GLMs with a *negative binomial 2* distribution and *log* link function, in which pollen deposition on L‐styled and S‐styled stigmas was treated as the response variable. Predictor variables included intra‐individual variation in corolla length, stigma length and stigma height.

Regarding pollen release from anthers, we fitted a generalized linear mixed model (GLMM) with a *binomial* distribution and a *logit* link function. The response variable was defined as pollen release from anthers, and an observation‐level random effect was included to account for overdispersion in the data (Harrison 2014). Predictor variables included the interaction between morphs and populations, as well as intra‐individual variation in corolla length and anther height. Our response variable (pollen release from the anthers) was calculated as the number of pollen grains in floral buds relative to the number remaining in anthers after pollinator visitation and combined using the *cbind* function (Crawley 2013). In addition, we assessed whether total pollen production differed between morphs and populations. We fitted a GLMM with a *Gamma* distribution and a *log* link function. The response variable was the estimated number of pollen grains produced per flower, while morph, population and their interaction were considered the predictor variables, with individual included as a random effect.

Regarding fruit set, we initially examined variations in fruit formation between morphs and populations before assessing the effects of intra‐individual variation. We fitted a generalized linear mixed model with a *binomial* distribution and a *logit* link function. The response variable was the proportion of fruit set, defined as the number of fruits formed relative to the total number of flowers, with these two variables combined using the *cbind* function (Crawley 2013). We incorporated an observation‐level random effect to account for overdispersion in the data (Harrison 2014). The predictor variables included morph, population and their interaction. We then investigated the influence of intra‐individual variation on fruit formation within each population, using a similar model, with the proportion of fruit set as the response variable. In the BSF population, predictor variables included intra‐individual variation in corolla length, stigma length and stigma height. In the PESCAN population, morph was also included as a predictor because fruit set differed between morphs in this population.

Lastly, we investigated whether intra‐individual variation was related to floral resource features, specifically nectar sugar and energy content. We initially explored variation in total sugar and energy content between morphs and populations using a GLM with a *Gaussian* distribution and *identity* link function. The response variable was total sugar and energy content per individual, and predictor variables included morph, population and their interaction. After detecting a significant effect (in this case, between populations), we assessed the relationship between total sugar and energy content and intra‐individual variation in the BSF and PESCAN populations. We again fitted a GLM with a *Gaussian* distribution and an *identity* link function, with total sugar per individual as the response variable, and corolla intra‐individual variation as the predictor.

We used the *glmmTMB* R package to fit all models (Brooks *et al*. 2017). We checked the models using QQ plots of residuals and predicted vs. residual plots, based on 250 simulated residuals generated with the DHARMa R package (version 0.4.5; Hartig & Lohse 2022). We applied a type III Wald Chi‐square test using the *car* R package to test the significance of the models (Fox *et al*. 2018). To report and plot results, we used the model‐adjusted values, back‐transformed with the *ggpredict* function from the *ggeffects* package in R (version 1.1.4; Lüdecke 2018). All analyses were performed using R software (R Core Team 2023).

## RESULTS

We found differences in intra‐individual variation at the stigma level between morphs and populations (Table S1). Intra‐individual variation in stigma length differed significantly between morphs (χ^2^ = 9.520, df = 1, *P* = 0.002, Table S1; Fig. 2A), with L‐styled flowers exhibiting 64% greater variation than those of S‐styled flowers. No significant differences in population or the morph × population interaction were detected for stigma length (Table S1; Fig. 2B,C). In contrast, intra‐individual variation in stigma height showed significant effects of morphs and the morph × population interaction (χ^2^ = 8.409, df = 1, *P* = 0.003 and χ^2^ = 4.548, df = 1, *P* < 0.032, respectively; Table S1; Fig. 2D,F). Stigma height variation was approximately 60% higher in S‐styled than in L‐styled flowers, but this pattern differed between populations, as indicated by a significant morph × population interaction. Specifically, in the BSF population, stigma height showed 60.1% greater intra‐individual variation in S‐styled than in L‐styled flowers, whereas no significant difference between morphs was detected in PESCAN. Population alone did not have a significant effect on intra‐individual variation in stigma height (Table S1; Fig. 2E). No significant effects of morph, population or their interaction were detected for intra‐individual variation in corolla length, anther length or anther height (Table S1).

**Fig. 2:**
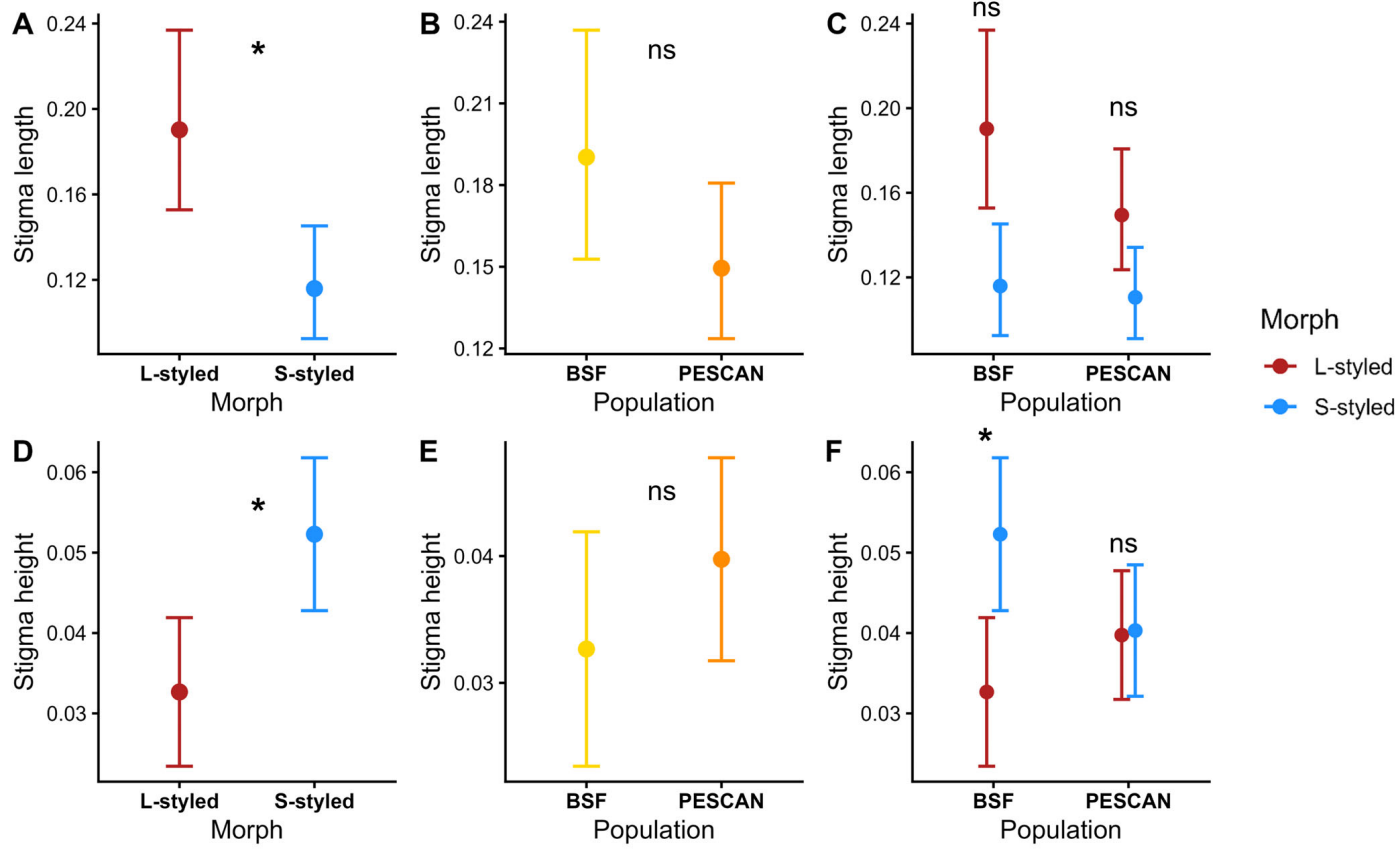
Intra‐individual variation in stigma length and stigma height in distylous *Palicourea rigida* populations is shown for (A, D) morphs, (B, E) populations and (C, F) interaction (morph × population). Points and line segments indicate back‐transformed predicted probabilities and 95% CIs, respectively. Asterisks indicate statistically significant differences at the 0.05 level; ‘ns’ denotes non‐significant effects.

We found significant effects of floral traits on pollen deposition on stigmas, with distinctions between morphs and populations (χ^2^ = 17.455, df = 1, *P* < 0.001 and χ^2^ = 6.403, df = 1, *P* = 0.011, respectively; Fig. 3A,B), whereas the morph × population interaction was not significant (χ^2^ = 2.10, df = 1, *P* = 0.146; Fig. 3C). L‐styled stigmas exhibited 134% higher pollen deposition than S‐styled stigmas. On average, the BSF population received 60% more pollen deposition than the PESCAN population. When we investigated whether the higher pollen deposition observed in the L‐styled morph was associated with intra‐individual variation within each population, we found a significant effect of corolla length in the BSF population (χ^2^ = 6.428, df = 1, *P* = 0.011; Fig. 4). Intra‐individual variation of corolla length showed a positive relationship with the number of pollen grains deposited on the stigmas of the L‐styled flowers, explaining 15% of the variance (Fig. 4). The other predictor variables, including intra‐individual variation in stigma length and stigma height, had no significant effects on pollen deposition in either the BSF or in PESCAN populations. When this analysis was performed for the S‐styled morph, which received fewer pollen grains, no significant effects were detected. Concerning pollen release from anthers, no significant effects were observed for morph, population, their interaction or intra‐individual variation in morphological traits. There were no significant effects of morph (χ^2^ = 0.53, df = 1, *P* = 0.466), population (χ^2^ = 0.85, df = 1, *P* = 0.357) or their interaction (χ^2^ = 0.48, df = 1, *P* = 0.487) on pollen production.

**Fig. 3:**
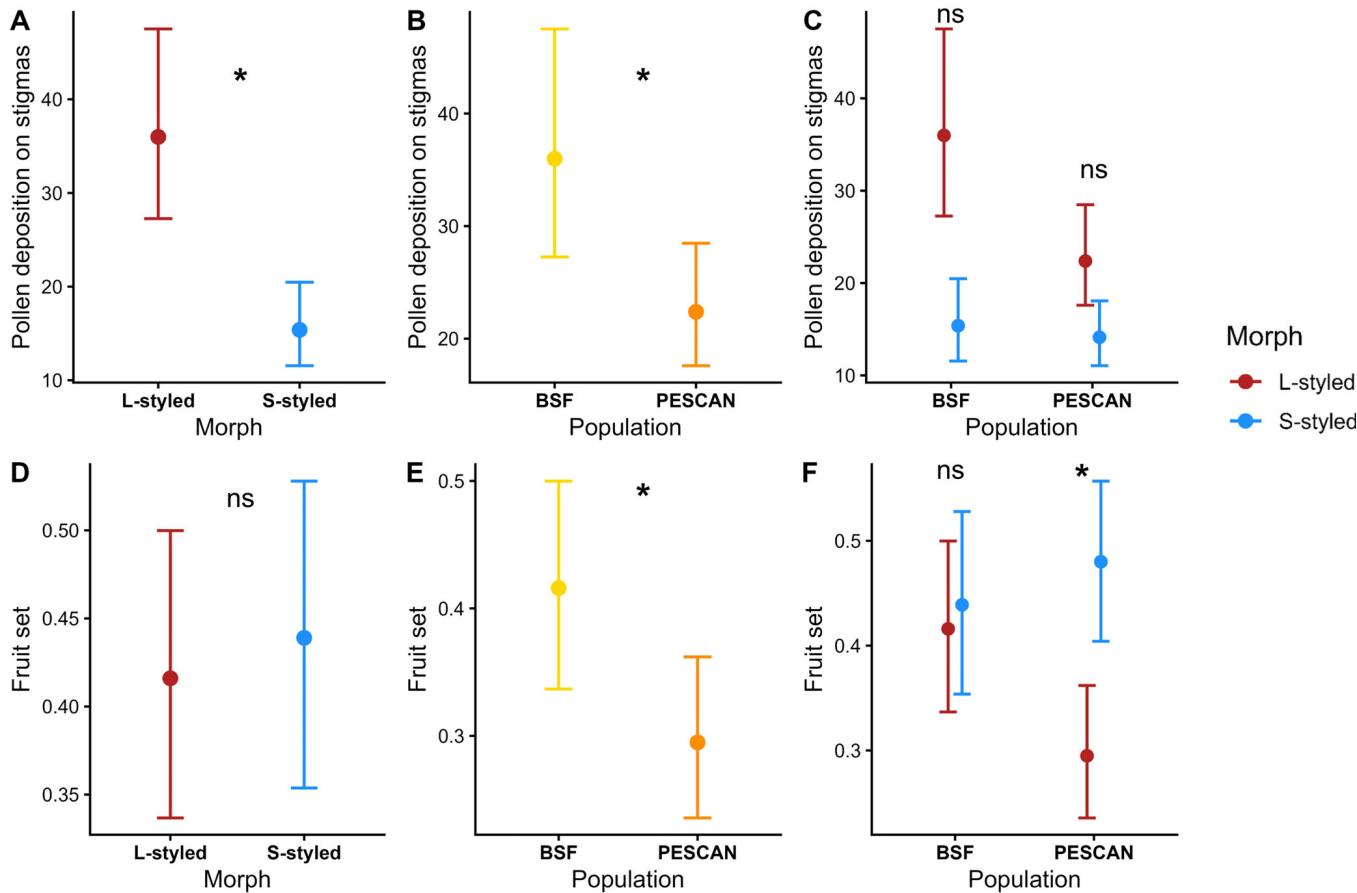
Pollen deposition on stigmas and fruit set (expressed as the proportion of flowers that developed into fruits) in distylous *Palicourea rigida* populations is shown for (A, D) morphs, (B, E) populations and (C, F) interaction (morph × population). Points and line segments indicate back‐transformed predicted probabilities and 95% CIs, respectively. Asterisks indicate statistically significant differences at the 0.05 level; ‘ns’ denotes non‐significant effects.

**Fig. 4:**
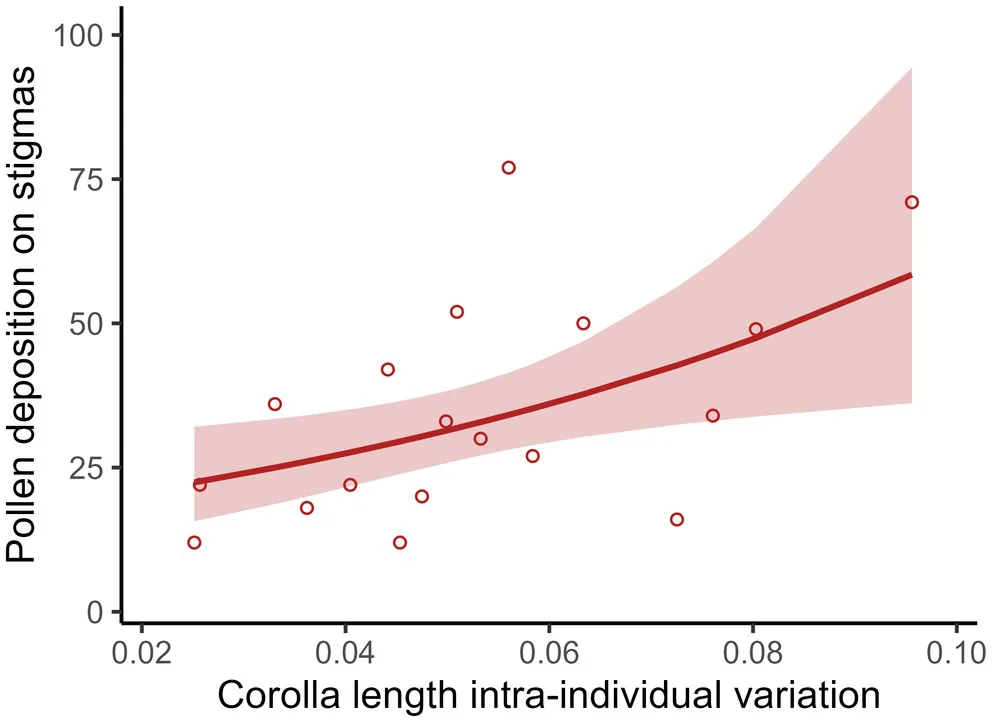
Positive effect of corolla length intra‐individual variation (predictor variable) on pollen deposition on stigmas in L‐styled flowers (response variable) in the BSF population. The line indicates the fitted model, red bands indicate the 95% confidence interval, and red points correspond to the raw data.

Significant effects were observed in the proportion of fruit set, with differences detected between populations and for the morph × population interaction (χ^2^ = 5.245, df = 1, *P* = 0.022 and χ^2^ = 4.35, df = 1, *P* = 0.037, respectively; Fig. 3E,F), whereas the effect of morph was not significant (χ^2^ = 0.139, df = 1, *P* = 0.708; Fig. 3D). The BSF population exhibited a fruit‐set 41% higher than the PESCAN population. Examination of the interaction term revealed that the effect of morph differed between populations. Specifically, in the PESCAN population, the S‐styled morph produced, on average, 62% more fruits than the L‐styled morph (Fig. 3F). In contrast, no significant differences between morphs were observed in the BSF population (Fig. 3F). When testing the effect of intra‐individual variation on fruit production within each population, no significant effects were observed.

A significant difference in nectar features was detected between populations (χ^2^ = 4.207, df = 1, *P* = 0.040; Fig. S3). On average, individuals from the BSF population had 52% higher total sugar and energy content than those from PESCAN. Neither morph nor the morph × population interaction had a significant effect, and no relationship was detected between nectar properties and intra‐individual variation in corolla length in either population.

## DISCUSSION

This study investigated the intra‐individual variation in floral traits and differences between morphs and populations in *Palicourea rigida*, a distylous plant with distinct pollinator groups. We investigated whether such variation had implications for reproductive success and nectar properties. Our results revealed that intra‐individual floral variation was not uniformly expressed across floral traits but was instead trait‐specific and context‐dependent. Intra‐individual variation differed between morphs and populations primarily at the stigma level (stigma length and stigma height). Regarding pollen deposition on stigmas, we found differences between morphs and populations, as well as a relationship with corolla length, highlighting the importance of morphological variation for pollination performance among individuals. Nevertheless, we found no effect of intra‐individual variation on fruit‐set or nectar features, despite differences between morphs and populations. These findings are discussed in detail below.

Intra‐individual variation in floral traits did not differ between morphs and populations of distylous *P. rigida*, except at the stigma level (stigma length and stigma height). This indicates that, overall, floral traits exhibit little to no intra‐individual differentiation between morphs, potentially reflecting constraints on the accuracy and functioning of distyly (Armbruster *et al*. 2017). However, intra‐individual variation in stigma length was higher in L‐styled flowers, a pattern consistent across populations. This is consistent with previous results for the species but differs from the trends reported for Rubiaceae as a whole, which showed greater intra‐individual variation in stigma length of S‐styled (Trevizan *et al*. 2024a). Greater intra‐individual variation in stigma length may not necessarily be negative for flower reproductive success. For example, greater variation in style deflexion has been shown to increase pollen deposition and to result in heavier seeds in *Passiflora incarnata* (Dai *et al*. 2016). Therefore, greater variation in stigma length among flowers could increase the quantity of pollen deposition, which may explain the higher pollen loads observed on L‐morph stigmas in our study. Additionally, these higher pollen loads may be explained by the L‐morph stigma exposed outside the floral tube, facilitating contact with pollinators and potentially enhancing pollen reception (Nishihiro *et al*. 2000).

Concerning intra‐individual variation in stigma height, the morph × population interaction indicates that the pattern of variation differed between populations. In BSF, S‐styled flowers showed greater intra‐individual variation in stigma height than L‐styled flowers, whereas no difference between morphs was detected in PESCAN. This population‐dependent pattern may reflect differences in pollinator assemblages that can exert distinct selective pressures on flower traits. The BSF population is mainly pollinated by large bees (Doria 2020), whereas the PESCAN population is pollinated by hummingbirds, attracting visits from various hummingbird species that differ in mass and bill length (see Justino *et al*. 2012). While variations in flower morphology at the population level are often associated with a decline in pollination efficiency in distylous plants (Ganders 1979), recent evidence suggests that flower variation at the individual level may actually enhance the chances of pollen deposition across flowers within the same individual plant (Dai *et al*. 2016; Pinto *et al*. 2022; Trevizan *et al*. 2024b). Pollinator groups with distinct morphological features may efficiently deposit pollen at different positions on the flower, targeting specific reproductive organs or heights. Such spatial heterogeneity in pollen placement may enhance the chances of legitimate pollen transfer among individuals, contributing to female fitness at the population level (Bronstein *et al*. 2006; Dai *et al*. 2016; Arceo‐Gómez *et al*. 2017).

Pollen deposition differed between morphs, being higher on L‐styled stigmas and in BSF than in PESCAN. We also found that higher intra‐individual variation in corolla length was associated with higher total pollen deposition on L‐styled stigmas in the BSF population. Studies have shown that flower size and shape can vary in relation to the morphology of the local pollinator assemblage (Nagano *et al*. 2014; Kuriya *et al*. 2015; Solís‐Montero & Vallejo‐Marín 2017; Ballarin *et al*. 2025). For plants with spatially segregated sexual organs and self‐incompatibility systems, such as distylous flowers, a proper fit between floral morphology and pollinator bodies is essential for intermorph disassortative pollination (Keller *et al*. 2014; Brys & Jacquemyn 2019). Because pollen is deposited and picked up on different parts of the pollinator's body, variation in corolla length may modulate how floral visitors contact stigmas and anthers, influencing pollen transfer efficiency between morphs (Nagano *et al*. 2014; Kuriya *et al*. 2015; Solís‐Montero & Vallejo‐Marín 2017). As we did not distinguish between self‐, intra‐ and intermorph pollen in our study, we cannot confirm whether this association reflects intermorph pollen transfer. Future research that combines analyses of pollen deposition with identification of pollen–morph origin could yield a more precise understanding of how variation in corolla length influences intermorph pollen transfer. This effect appears particularly noticeable for L‐styled flowers, where the stigma is positioned above the anthers. However, it is worth noting that the lower pollen deposition in S‐styled flowers was not influenced by intra‐individual variation, suggesting morph‐specific constraints or differences in pollinator contact. Additionally, in this study, the total pollen deposited likely contains self‐ and intramorph pollen, both of which can impair female fitness by causing, for example, stigma clogging (Bertin 1993).

Intra‐individual variation was not linked to fruit production or nectar properties, despite differences between morphs and populations. One possible explanation is that flowers have only two ovules (Machado *et al*. 2010), so even low pollen deposition may be sufficient for fertilization, which may explain the lack of a relationship between intra‐individual variation and fruit set. Regarding nectar properties, the BSF population exhibited higher total sugar and energy content than the PESCAN population. This aligns with local adaptations, as hummingbirds and bees differ in their energetic demands and feeding physiology, which can select for differences in nectar components (Castellanos *et al*. 2004; Izquierdo *et al*. 2023). Generally, bee‐pollinated flowers show more concentrated nectar than bird‐pollinated flowers (Fenster *et al*. 2004).

In conclusion, the absence of differences in intra‐individual variation in most floral traits between morphs suggests that similar levels of variation are maintained across morphs, potentially facilitating effective pollen transfer in self‐incompatible distylous populations. The differences in intra‐individual variation at the stigma level between morphs and populations may reflect contrasting pollination pressures, as one population is predominantly pollinated by hummingbirds and the other by bees. Although intra‐individual variation did not influence fruit production, likely due to the low number of ovules, corolla length showed a positive, morph‐dependent effect on pollen deposition regardless of pollen type (self/intra‐ or intermorph), particularly in L‐styled flowers from the BSF population. These findings provide a new perspective on the role of intra‐individual variation in the reproduction of distylous plants, suggesting it may enhance pollination performance by allowing flowers to exploit a broader positional range on pollinators, thereby increasing cross‐pollen deposition among flowers within individuals.

## AUTHOR CONTRIBUTIONS

RT, FT and PEO planned and designed the research. Data collection was done by RT, FT, LM, LO and FA. Analysis and writing of the first manuscript draft were done by RT, PEO and VF. Figures were done by RT, LO and FA. All authors read, provided suggestions and approved the final version of the manuscript.

## CONFLICT OF INTEREST STATEMENT

The authors declare no conflicts of interest.

## Supporting information


**Fig. S1.** Geographic location of the two studied *Palicourea rigida* populations: Caldas Novas State Park (PESCAN), Caldas Novas, Goiás, Brazil, and Botucatu State Forest (BSF), Botucatu, São Paulo, Brazil;

**Fig. S2.** Illustration of floral morphs: long‐style (L‐styled) ‐ stigma above anthers, and short‐style (S‐styled) ‐ stigma below anthers, positioning of high and low organs, and representation of morphological measurements taken: (1) corolla length; (2) stigma length; (3) stigma height; (4) anther length; and (5) anther height. (Illustration by Ricardo Ribeiro da Silva);

**Fig. S3.** Total nectar sugar (mg) and energy content showing differences between populations. Points and line segments indicate back‐transformed predicted probabilities and 95% CIs, respectively. Asterisks indicate significant differences at the 0.05 level.

**Table S1.** Results of generalized linear models (GLMs) investigating intra‐individual variation in floral morphological traits between morphs, populations, and their interaction (Morph × Population) in *Palicourea rigida*.

## Data Availability

The data that support the findings of this study are available on request from the corresponding author. The data are not publicly available due to privacy or ethical restrictions.
