# Supplementary material for: Floral intra‐individual variation differs between morphs and is linked to pollen deposition in self‐incompatible distylous plants
Source: Plant Biol (Stuttg). 2026 Sep 25;28(7):2327–36. doi: 10.1111/plb.70299 (PMC13750339; doi:10.1111/plb.70299)
Supplement: Supplementary file 1 [file PLB-28-2327-s003.docx]

**Supplementary Table**

**Table S1.** Results of generalized linear models (GLMs) investigating intra-individual variation in floral morphological traits between morphs, populations, and their interaction (Morph × Population) in *Palicourea rigida*. The table reports χ² values, degrees of freedom (df), and *p*-values. Significant p-values in bold.

| Response variable | Family | Morph | | |  | Population | | |  | Interaction | | |
| --- | --- | --- | --- | --- | --- | --- | --- | --- | --- | --- | --- | --- |
|  |  | χ^2^ | df | p |  | χ^2^ | df | p |  | χ^2^ | df | p |
| Corolla length | Gamma | 1.473 | 1 | 0.224 |  | 0.318 | 1 | 0.572 |  | 0.092 | 1 | 0.761 |
| Stigma length | Gamma | 9.520 | 1 | **0.002** |  | 2.264 | 1 | 0.103 |  | 0.834 | 1 | 0.361 |
| Stigma height | Gaussian | 8.409 | 1 | **0.003** |  | 1.287 | 1 | 0.256 |  | 4.548 | 1 | **0.032** |
| Anther length | Gamma | 0.910 | 1 | 0.339 |  | 2.438 | 1 | 0.118 |  | 0.655 | 1 | 0.418 |
| Anther height | Gamma | 0.088 | 1 | 0.765 |  | 0.376 | 1 | 0.539 |  | 0.074 | 1 | 0.784 |
